# Genetic variants determining survival and fertility in an adverse African environment: a population-based large-scale candidate gene association study

**DOI:** 10.18632/aging.100986

**Published:** 2016-06-27

**Authors:** Jacob J.E. Koopman, Jeroen Pijpe, Stefan Böhringer, David van Bodegom, Ulrika K. Eriksson, Hernando Sanchez-Faddeev, Juventus B. Ziem, Bas Zwaan, P. Eline Slagboom, Peter de Knijff, Rudi G.J. Westendorp

**Affiliations:** ^1^ Section of Gerontology and Geriatrics, Department of Internal Medicine, Leiden University Medical Center, Leiden, the Netherlands; ^2^ Department of Human Genetics, Leiden University Medical Center, Leiden, the Netherlands; ^3^ University of Applied Sciences Leiden, Leiden, the Netherlands; ^4^ Section of Medical Statistics, Department of Medical Statistics and Bioinformatics, Leiden University Medical Center, Leiden, the Netherlands; ^5^ Leyden Academy on Vitality and Ageing, Leiden, the Netherlands; ^6^ Department of Clinical Laboratory Sciences, School of Medicine and Health Sciences, University for Development Studies, Tamale, Ghana; ^7^ Laboratory of Genetics, Wageningen University, Wageningen, the Netherlands; ^8^ Section of Molecular Epidemiology, Department of Medical Statistics and Bioinformatics, Leiden University Medical Center, Leiden, the Netherlands; ^9^ Department of Public Health and Center of Healthy Aging, University of Copenhagen, Copenhagen, Denmark

**Keywords:** Africa, aging, evolution, fertility, gene, human, life history, SNP, survival

## Abstract

Human survival probability and fertility decline strongly with age. These life history traits have been shaped by evolution. However, research has failed to uncover a consistent genetic determination of variation in survival and fertility. As an explanation, such genetic determinants have been selected in adverse environments, in which humans have lived during most of their history, but are almost exclusively studied in populations in modern affluent environments. Here, we present a large-scale candidate gene association study in a rural African population living in an adverse environment. In 4387 individuals, we studied 4052 SNPs in 148 genes that have previously been identified as possible determinants of survival or fertility in animals or humans. We studied their associations with survival comparing newborns, middle-age adults, and old individuals. In women, we assessed their associations with reported and observed numbers of children. We found no statistically significant associations of these SNPs with survival between the three age groups nor with women's reported and observed fertility. Population stratification was unlikely to explain these results. Apart from a lack of power, we hypothesise that genetic heterogeneity of complex phenotypes and gene-environment interactions prevent the identification of genetic variants explaining variation in survival and fertility in humans.

## INTRODUCTION

Age patterns of survival and fertility vary widely across species [1]. During evolution, natural selection has shaped these age patterns, referred to as life histories, so to optimise the fitness of each species by maximising reproduction [2]. Reproduction is increased if survival or fertility are enhanced or if a decline in survival or fertility with age is resisted. The diversity in life history across species indicates that it has a strong genetic basis. Several genetic pathways have been found in animals that regulate survival and fertility, including the signalling cascade of growth hormone (GH), insulin-like growth factor 1 (IGF1), and insulin, signalling by target of rapamycin (TOR), DNA repair mechanisms, immune regulation, and telomere maintenance [3, 4]. These pathways have been discovered mostly in studies on mutant animal models, but likely contribute to variation in survival and fertility in wild-type animals as well [5, 6]. Genetic variation in these pathways is thought to likewise determine the age patterns of survival and fertility in humans [7], but it remains disappointingly inconclusive as an explanation of observed variation in human survival and fertility. Candidate-gene studies, linkage studies, and genome-wide association studies have yielded consistent evidence for only a handful of genetic variants to determine variation in human survival to old age, of which most notably *APOE* [8–11]. Genetic variants have been described as determinants of human infertility [12, 13], but have rarely been studied for variation in human fertility [14].

Research on the genetic determinants of human life history has almost exclusively been conducted in populations living in modern affluent environments. These modern affluent environments, however, are radically different from the environments in which over many generations humans have been subjected to evolutionary pressures. During most of human history, survival and fertility were compromised by infectious diseases [15, 16], malnutrition [17, 18], climatic hardships, predation, and violence [19]. Fitness, which includes survival and fertility, has long been shaped by natural selection enforced through these environmental stressors. It is, therefore, likely that natural selection has enhanced survival and fertility by promoting genetic variants that shape inflammatory processes to improve resistance against infections, metabolic processes to facilitate consumption and storage of nutrients, and psychological strategies to cope with environmental stressors. In modern affluent environments, however, where survival and fertility depend less on these adverse environmental stressors, such genetic variants are of less influence on life history.

If we aim to identify the genetic determinants of life history that have enhanced fitness in adverse environments, we should search for them in such environments. In this study, we investigate genetic variants that determine life history through variation in survival and fertility in a traditional rural African population that lives in such an adverse environment. Compared with modern affluent environments, this population's mortality rates and fertility rates are high, various infectious diseases are endemic, periodic food shortage and malnutrition are common, and a sedentary lifestyle is absent [20–29].

## RESULTS

Table 1 shows the general characteristics of the Ghanaian study population. Genetic variants as determinants of variation in survival were investigated in men and women together, grouped as newborns, middle-aged adults of fertile ages from 20 through 44 years, and old individuals aged 60 years or over. Genetic variants as determinants of variation in fertility were investigated in women only. Observed fertility was registered in middle-aged women of fertile ages from 20 through 44 years during follow-up. Reported fertility was registered in postmenopausal women aged 45 years or over at the beginning of follow-up.

**Table 1:** General characteristics of the Ghanaian study population

	Men and women			Women	
	0 years	20-44 years	≥ 60 years	20-44 years	≥ 45 years
Individuals, *n*	1482	1589	1144	732	708
Females, *n* (%)	695 (46.9)	1394 (87.7)	608 (53.1)	732 (100.0)	708 (100.0)
Age, years	0 (0–0)	33 (26–40)	70 (65–77)	33 (27–37)	63 (56–71)
Tribe, *n* (%)					
Bimoba	1017 (68.6)	1124 (70.7)	696 (60.8)	549 (75.0)	446 (63.0)
Kusasi	367 (24.8)	365 (23.0)	357 (31.2)	142 (19.4)	205 (29.0)
Other	98 (6.6)	100 (6.3)	91 (8.0)	41 (5.6)	57 (8.1)
Observed fertility	NA	NA	NA	1 (1–2)	NA
Reported fertility	NA	NA	NA	NA	8 (6–9)

Data are given as numbers with percentages or as medians with interquartile ranges. Observed fertility is expressed as the number of children that a woman gave birth to during the period of follow-up. Reported fertility is expressed as the number of children that a woman had given birth to during life. NA: not applicable.

**Figure 1:**
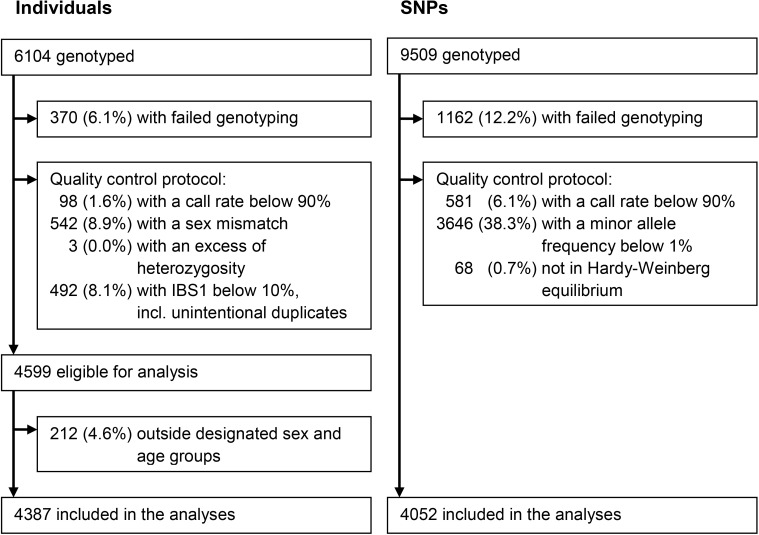
Summary of the exclusions and inclusions of individuals and SNPs

As shown in Figure 1, after the quality control 4052 SNPs, encompassing 148 genes, were included in the analyses. The median (interquartile range) number of kb between SNPs included in the analyses was 2.3 kb (1.8-3.4 kb). An overview of the included genes is given in Supplementary Table 1.

**Figure 2:**
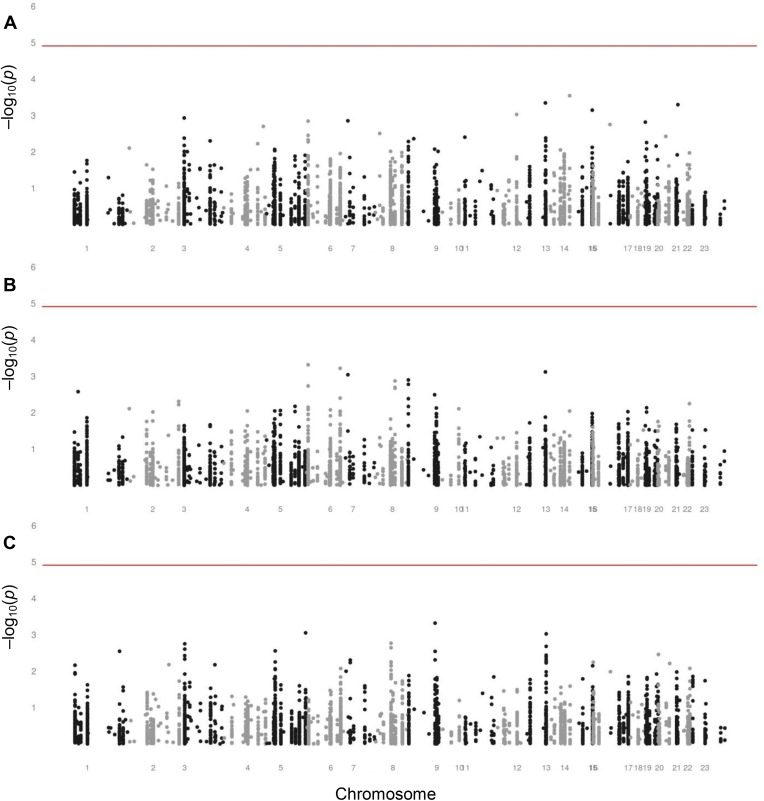
Manhattan plots assessing the associations of SNPs with survival (**A**) Manhattan plot assessing the associations of SNPs with survival between newborns and old individuals aged 60 years or over. (**B**) Manhattan plot assessing the associations of SNPs with survival between newborns and middle-aged adults of fertile ages from 20 through 44 years. (**C**) Manhattan plot assessing the associations of SNPs with survival between middle-aged adults of fertile ages from 20 through 44 years and old individuals aged 60 years or over. The analyses were adjusted for sex. The level of significance is 1.23 × 10^−5^, indicated by the red lines.

Figure 2 reports on the investigation of the genetic variants as determinants of variation in survival. We assessed the association of each SNP with the chance of being an old individual as compared with a newborn, a middle-aged adult as compared with a newborn, and an old individual as compared with a middle-aged adult, reflecting the survival between each pair of age groups. None of the SNPs were statistically significantly associated with survival between these three age groups.

Although each was non-significant, we list the ten SNPs with the lowest *p* values for the association with survival between each pair of age groups in Supplementary Tables 2, 3, and 4. SNPs in many different genes appeared in these lists, among which two neutrally selected control SNPs in the list comparing newborns and old individuals. None of these SNPs appeared in more than one of these lists, except for rs2026816 in the insulin receptor substrate 2 gene (*IRS2*) and rs2069842 in the interleukin 6 gene (*IL6*), both of which were in the lists comparing newborns and old individuals and comparing newborns and middle-aged adults.

**Figure 3:**
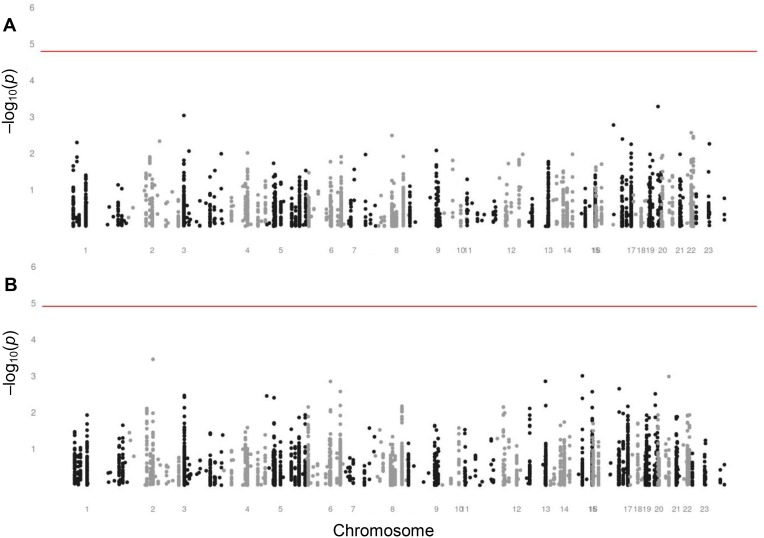
Manhattan plots assessing the associations of SNPs with fertility in women (**A**) Manhattan plot assessing the associations of SNPs with observed fertility in middle-aged women of fertile ages from 20 through 44 years. The level of significance is 1.61 × 10^−5^, indicated by the red line. (**B**) Manhattan plot assessing the associations of SNPs with reported fertility in postmenopausal women aged 45 years and older. The level of significance is 1.23 × 10^−5^, indicated by the red line.

Figure 3 reports on the investigation of the genetic variants as determinants of variation in fertility. We assessed the associations of each SNP with observed fertility in middle-aged women and with reported fertility in postmenopausal women. None of the SNPs were statistically significantly associated with either observed or reported fertility.

Although each was non-significant, we list the ten SNPs with the lowest *p* values for the associations with observed and reported fertility in Supplementary Tables 5 and 6. SNPs in many different genes appeared in these lists. Among them was a neutrally selected control SNP in the list for the association with observed fertility in middle-aged women. None of the SNPs appeared in both lists.

## DISCUSSION

The aim of this study was to identify the genetic variants that determine life history through variation in survival and fertility in a traditional rural African population that lives in an adverse environment without a western lifestyle. We conducted a large-scale candidate gene study using a high density of SNPs. We found no statistically significant association of any genetic variant with either survival or fertility.

Studies on the genetic determinants of variation in human survival and fertility in adverse environments have never been executed with numbers of individuals and SNPs comparable to this study. Due to a lack of previous genetic studies in this region, we could not rely on standard genome-wide association analyses given the higher degree of population stratification and the lesser degree of linkage disequilibrium in African populations [30]. Instead, we used a custom-made array for geno-typing a high density of SNPs in the genes of interest. A relatively large proportion of SNPs failed genotyping or had an insufficient minor allele frequency. The exceptional circumstances that are inherent to adverse environments – such as an inadequate infrastructure, a missing civil registry, and language and culture barriers – compromised the study's execution. The proportion of individuals with failed genotyping was comparable with studies in western affluent environments, but we rigidly excluded a relatively large proportion that appeared to have a mismatch between their genetic and registered sexes or to be unintentionally duplicated. To enlarge the contrast in the analysis of the SNPs' associations with survival the selection of old individuals could have been restricted to higher ages, but this would have diminished the number of included old individuals and the power of the analysis. A post hoc calculation of the present study's power, based on the numbers of individuals and SNPs included in the analyses after the quality control using an additive logistic regression model, reveals that we could find statistically significant associations with an odds ratio of at least 1.4 with a power of 80% and a minimal minor allele frequency ranging from 0.08 to 0.29 (Supplementary Figure 1). As we did not find such statistically significant associations, the associations are possibly present, but less strong.

Population stratification was unlikely to explain our results. As a result of the polygynous and patrilocal culture in the research area, men preferentially marry women from outside the region. Previous analyses of this study population have confirmed that the female-mediated gene flow is nearly fully random and prevents population stratification of autosomal genes [31]. To account for possible population stratification in this study, we rigidly excluded individuals that had a different allele at less than 10% of the loci indicated by an IBS1 below 10% and SNPs that were not in Hardy-Weinberg equilibrium. In addition, we adjusted our analyses for tribe, which represents a mainly cultural population stratification, and for the main dimensions of the multidimensional scaling (MDS) analysis, which represents a mainly genetic population stratification. The MDS analysis did not reveal any population stratification. The adjustments did not affect our results.

Here we did not find genetic variants that were statistically significantly associated with variation in survival or fertility. This finding corresponds with the difficulty to identify such variants in modern affluent environments. In such environments, variation in human lifespan is genetically determined for only less than 30%. Many and various studies have yielded consistent evidence for only a handful of genetic variants to determine variation in survival, most notably *APOE*, and most of their associations with survival are not strong [8–10]. Genetic variants that determine infertility have been identified, but genetic variants that determine variation in fertility have rarely been reported [14]. These disappointing results have been explained by the proposition that survival and fertility are determined by rare genetic variants with a strong effect or by a complex of interacting genetic variants with small effects. Both remain undetected in genome-wide or large-scale candidate gene association studies, such as our study [10, 11].

Contrary to many studies in affluent populations, the present study did not identify *APOE* as a determinant of variation in survival. Three SNPs in *APOE* were included in the present analyses: rs1081101, rs877973, and rs769450. The *p* values for their associations with survival ranged between 0.36 and 1.00. In West Africans, only the latter of these SNPs is in linkage disequilibrium (*r*^2^=0.21) with rs429358, the SNP that constitutes the *APOEε4* allele and most consistently determines variation in survival in modern affluent populations, but contrary to rs429358, it does not influence blood lipid levels. The other two SNPs are not in linkage disequilibrium with rs429358 and influence blood triglyceride, but not cholesterol levels [32].

Our finding that genetic variants known to influence survival in modern affluent populations, such as *APOE*, did not influence survival in adverse environments may likely be explained, apart from chance, by gene-environment interaction. The effects of genes vary depending on environmental conditions, which thus determine the effects of genes on survival and fertility [33]. Our study was conducted in an environment characterised by endemic infectious diseases, shortages of food, necessary physical activity, and a scarcity of cardiovascular disease and diabetes up to high ages [20–29]. These characteristics differ radically from those of modern affluent environments. Variants of *APOE* affect blood lipid levels, the risk of cardiovascular disease, and survival in affluent populations [34]. In this study population, lipid levels as well as the risk of cardiovascular disease are far lower than in affluent populations [25–28], which may explain why *APOE* does not affect survival here. The higher levels of physical activity may provide an additional explanation for the absence of such an effect [35]. Likewise, we have previously shown that variants of *IL10*, associated with the inflammatory strength of the immune response, enhance survival in those exposed to contaminated drinking water, but diminish survival in those exposed to clean drinking water [36]. These examples of different effects of genetic variants in different environments indicate that such effects can only be identified if, firstly, a specific hypothesis is formulated, secondly, the environmental conditions are measured with the same rigour as the genetic variants, and, thirdly, corresponding appropriate statistical methods are applied.

Life histories have evolved as natural selection has optimised fitness by increasing the frequencies of genetic variants that enhance survival and fertility, while decreasing the frequencies of genetic variants that diminish survival and fertility. The effects of genetic variants on survival and fertility, and thereby the evolution of life history, are largely dependent on environmental factors [2]. The diversity in life history across species indicates that it has a genetic basis, but the diversity in the genetic variants that determine variation in survival and fertility across populations and environ-ments within species indicates that these genetic variants differ across species, populations, and environments.

Evolutionary theory predicts that most of the genetic variants that enhance survival diminish fertility or vice versa, because a trade-off exists between investments in survival and investments in fertility [37, 38]. Indeed, genetic variants have been described that influence both survival and fertility [39, 40]. The close relation between survival and fertility during evolution has encouraged us to study them jointly. Furthermore, several evolutionary theories explain why survival and fertility decline with age in humans and many other species. According to one theory, random damage accumulates in the genetic determinants of survival and fertility as natural selection loses its strength [37, 38]. Such genetic damage as determinants of life history that differ across individuals cannot be identified by a study like the present. According to another theory, some genetic determinants that enhance survival and fertility at early ages have an antagonistic pleiotropic effects that diminish survival and fertility at later ages [37, 38]. The present study would have been able to identify such genetic determinants.

It is critical to acknowledge that natural selection works to maximize fitness as a phenotype rather than a genotype, with survival and fertility being the most important components of this phenotype. The genotype only matters to natural selection as a determinant of the phenotype that is under selection. Meanwhile, the phenotype may be the result of a complex interaction between various genetic variants. Natural selection exists by virtue of such variation in the genetic determinants. Genetic variation is conserved, since genes are prone to mutations and new genetic variants are introduced by sexual reproduction. The resulting genetic heterogeneity of populations may explain why so few unique genetic variants have been found to determine variation in the complex phenotypes of survival and fertility. If different genetic variants determine a similar phenotype, each of these genetic variants is shared by only a proportion of the individuals and a patchwork of shared and unshared variants is established throughout the population. Moreover, if different genetic variants interact with each other to express a phenotype, the penetrance of one of these genetic variants relative to another may vary throughout the population. When studied in a population as a whole, these patterns lead to annulation of the effect of a single genetic variant. As an example of genetic heterogeneity underlying a single phenotype, the ability to digest milk after childhood due to lactase persistence has independently evolved multiple times in similar environments of animal domestication. Across populations, various SNPs at different positions in the lactase gene bring about this trait. Still, these specific variants are insufficient to explain differences in the frequencies of lactase persistence across populations [41, 42]. Methods to analyse multiple interacting genetic variants are possible, but beyond the aim of this study. More fundamentally, the complexity of the genotype to give rise to a phenotype suggests that, next to explaining genetic variation in survival and fertility, it may be more worthwhile to search for the causal biological mechanisms that determine survival and fertility.

In conclusion, we aimed to identify the genetic variants that determine life history through variation in survival and fertility in an adverse environment in rural Africa, which resembles the environments during most of recent human evolution. In this large-scale candidate gene study, we did not find statistically significant associations of genetic variants with survival or fertility. Apart from a lack of power, we hypothesise that genetic heterogeneity of complex phenotypes and gene-environment interaction prevent the identification of such unique genetic variants that humans have been selected for.

## METHODS

### Study population

This study was conducted in the Garu-Tempane District in the Upper East Region in Ghana. The region is rural, remote, and one of the least developed in the country. The vast majority of the inhabitants are involved in subsistence agriculture performed by manual labour without proper means of transportation or mechanized farming. The mean annual per capita income and expenditure in the region are one third of those in Ghana nationally and one fifth of those in the capital Accra [43]. Of the adult inhabitants, 31% has attended school as compared with 69% in Ghana nationally and 89% in the capital Accra [43]. Hospital care is absent. Various infectious diseases – including malaria, measles, meningitis, tuberculosis, typhoid fever, trachoma, and intestinal helminths – are highly endemic and constitute the main causes of death both in childhood and adulthood, although the prevalence of human immunodeficiency virus (HIV) is low (<4%) compared with other African regions [44].

From 2002 through 2011, we kept a demographic registry of the population in a research area of 375 km^2^ comprising 32 villages. During annual visits we registered the name, age, sex, tribe, and location of living of each inhabitant. If an inhabitant's age was unknown, it was estimated by oral methods, as described previously [20, 21]. Households were occupied by extended families with 48% of the married men having multiple wives [45]. Annual migration relative to the study population's size was 2% into and 1% out of the research area. The average property of the households included small numbers of cattle and bicycles with a value of circa 1,000 US$ and 15% of the households had access to electricity [22, 45]. Drinking water was drawn from boreholes, open wells, and rivers [36]. Of apparently healthy adults, 86% were infected by the malaria species *Plasmodium falciparum*, 44% by the protozoan *Giardia lamblia*, and 31% by the helminth *Necator americanus* [24]. During the nine years of follow-up, 46 to 53% of the population was aged less than 15 years and 6 to 7% of the population was aged 60 years and more [28].

Ethical approval was given by the Committee Medical Ethics of the Leiden University Medical Center, the Ethical Review Committee of Ghana Health Services, and the local chiefs and elders. Because of illiteracy, informed consent was obtained orally from the participants after explanation of the purpose and conduction of this research project. The data were analysed anonymously. This study was conducted in accordance with the Declaration of Helsinki.

### Survival and fertility

Survival of all inhabitants was registered during the annual follow-up from 2002 through 2011 [28]. Fertility of women was measured through their reproduction in two manners. Firstly, observed fertility was registered prospectively for women of all ages during the annual follow-up from 2002 through 2011. Observed fertility was expressed as the number of children that a woman gave birth to during the period of follow-up [28, 46]. Secondly, reported fertility was retrospectively determined in 2003 by interviewing women who were available and willing to participate. Data on reported fertility was restricted to women aged 45 years and older, who were considered to be postmenopausal and for whom reported fertility represented lifelong reproduction. Reported fertility was expressed as the number of children that a woman had given birth to during life [21, 47].

### DNA collection, isolation, processing, and genotyping

To identify genetic variants associated with survival, we aimed to contrast the genotypes of newborns, of middle-aged individuals, and of individuals who had survived to old age. To identify genetic variants associated with fertility, we aimed to associate the genotypes of middle-aged and postmenopausal women with their reported or observed fertility. Since 2003, we took buccal samples from all newborns who were present during our visits, older than one week, and born in the same year. We took buccal samples from men and women of the fertile ages from 20 through 44 years, from women aged 45 years and older, and from men and women aged 60 years and older. The buccal samples of middle-aged and older individuals were collected together with measurements of phenotypic characteristics, including fertility [21, 47], infectious diseases [24], inflammatory and metabolic markers in blood [23, 25, 26, 36], cardiovascular health [25–27], muscle strength [29, 48]. These characteristics had been measured in randomly selected individuals. In addition, we collected buccal samples in 2010 from individuals randomly selected from our demographic registry to obtain balanced numbers in the three age groups. With an eye to the analyses of fertility, we oversampled middle-aged women over men.

The buccal samples were stored in 2.5 ml STE buffer (100 mM NaCl, 10 mM Tris/HCl, 10 mM EDTA, pH 8.0) with 0.05 mg/ml proteinase K, 0.1 mg/ml pronase, and 0.5% sodium dodecylsulphate. DNA was isolated and processed from samples collected in 2002 through 2006 by BaseClear (Leiden, the Netherlands) using a Chemagic bead-extraction method, from samples collected in 2006 through 2008 by the Department of Molecular Epidemiology of Leiden University Medical Center (Leiden, the Netherlands) using Qiagen silica spin-columns, and from samples collected in 2007 through 2011 by LGC Genomics, formerly KBioscience (Middlesex, UK), using a proprietary silica column method. DNA was genotyped using a custom-made Illumina Infinium iSelect High-Density Custom Genotyping BeadChip (Illumina, San Diego, CA) at the Department of Human Genetics of Leiden University Medical Center following the manufacturer's instructions.

### Candidate gene selection

For the genotyping and analyses, we selected 153 candidate genes that were considered relevant for regulation of life history based on literature, discussion with experts, and general inference. These candidate genes included genes associated with survival or fertility in genome-wide association studies in humans or model organisms [49], human genes that have been under positive or balancing selection pressure during the last 100,000 years and that are associated with survival or fertility [15, 50, 51], and homologues of genes associated with survival in model organisms [52].

### SNP selection

We aimed for a dense coverage of SNPs in the candidate genes. We selected SNPs that are known to be causally associated with survival or fertility in humans, had a minor allele frequency equal to or higher than 1% and a correlation equal to or higher than 0.8 in the Yoruba population [53], and/or had a genotype score higher than 0.8 according to the Illumina Design Tool (Illumina, San Diego, CA). If necessary, we chose tag SNPs using Tagger [54] and/or OpenHelix Genome Variation Server [55] based on the Yoruba population [53]. We additionally selected 170 control SNPs that are presumed to be selectively neutral for analysis of population stratification [56, 57]. The median (interquartile range) number of 1000 base pairs (kb) between genotyped SNPs was 0.8 kb (0.8-1.0 kb).

### Quality control

After genotyping, buccal samples and SNPs were subjected to a stringent quality control protocol. Of the 6104 individuals from whom buccal samples had been taken, we excluded 370 (6.1%) because of genotyping failure. Furthermore, we excluded 98 (1.6%) individuals with a call rate below 90%, 542 (8.9%) individuals with a sex mismatch, 3 (< 0.1%) individuals with an excess of heterozygosity indicated by an inbreeding coefficient below −0.3 or above 0.3, and 492 (8.1%) individuals with a different allele at less than 10% of the loci indicated by an IBS1 below 10%, which included unintentional duplicates. Of the 4599 individuals eligible for analysis, 212 (4.6%) individuals outside the designated sex and age groups were excluded. Of the 9509 genotyped SNPs, we excluded 1162 (12.2%) because of genotyping failure. Furthermore, we excluded 581 (6.1%) SNPs with a call rate below 90%, 3646 (38.3%) SNPs with a minor allele frequency below 1%, and 68 (0.7%) SNPs that were not in Hardy-Weinberg equilibrium. As a result, 4387 individuals and 4052 SNPs were included in the analyses. Of these SNPs, 98 (2.4%) were evolutionarily neutrally selected controls. The exclusions and inclusions are described in Figure 1.

As previously reported for this study population, population stratification is unlikely to influence any associations with genetic variation in autosomal genes. As a result of the polygynous and patrilocal culture in the research area, the female-mediated gene flow is nearly fully random [31]. Multidimensional scaling (MDS) analysis did not reveal any population stratification. Potential residual population stratification was addressed in the analyses.

### Analyses

For the investigation of the genetic determinants of survival, we assessed the association of each SNP with the chance of being in one of three age groups: newborns, middle-aged adults of fertile ages from 20 through 44 years, and old individuals aged 60 years or over. We compared the chances between pairs of these groups using an additive logistic regression model adjusted for sex. For the investigation of the genetic determinants of fertility, we assessed the associations of each SNP with observed fertility in middle-aged women of fertile ages from 20 through 44 years and with reported fertility in postmenopausal women aged 45 years and older. We assessed the association with reported fertility using an additive linear regression model. We assessed the association with observed fertility using an Andersen-Gill model, which is an extension to the Cox regression model for analysis of recurrent events [58]. The model was adjusted for calendar year in order to account for the decline in fertility observed during the period of follow-up [28]. To avoid convergence of this model it was necessary to exclude 954 SNPs with a minor allele frequency below 5% from this assessment. All models were repeated with additional adjustment for tribe as a categorical covariate, with additional adjustment for the first two dimensions of the MDS analysis, with additional clustering per household, or with a combination of these in order to account for potential population stratification. These additional adjustments did not alter the results. After a Bonferroni correction for the number of SNPs included in the analyses the threshold for significant results was set at 1.23 × 10^−5^ for the logistic and linear regression models and at 1.61 × 10^−5^ for the Andersen-Gill model. The analyses were performed using R (R Foundation, Vienna, Austria).

## SUPPLEMENTARY DATA TABLES AND FIGURES


