# Supplementary material for: Genetic variants determining survival and fertility in an adverse African environment: a population-based large-scale candidate gene association study
Source: Aging (Albany NY). 2016 Jun 27;8(7):1364–74. doi: 10.18632/aging.100986 (PMC4993336; doi:10.18632/aging.100986)
Supplement: Supplementary file 1 [file aging-08-1364-s001.pdf]

SUPPLEMENTARY DATA

Supplementary Table 1. Candidate genes included in the analyses

Chromo-some	Gene symbol	Gene name	Number of SNPs	Average kb per SNP
14	*AKT1*	V-akt murine thymoma viral oncogene homolog 1	10	3.1
19	*AKT1S1*	AKT1 substrate 1	7	1.9
22	*APOBEC3G*	Apolipoprotein B mRNA editing enzyme catalytic polypeptide-like 3G	4	3.9
11	*APOC3*	Apolipoprotein C-III	5	1.6
19	*APOE*	Apolipoprotein E	3	2.9
X	*AR*	Androgen receptor	36	5.2
13	*ARHGEF7*	Rho guanine nucleotide exchange factor 7	98	2.0
1	*ARID1A*	AT rich interactive domain 1A	21	4.3
21	*BACH1*	BTB and CNC homology 1, basic leucine zipper transcription factor 1	73	4.5
19	*BRSK1*	BR serine/threonine kinase 1	11	3.0
16	*CASKIN1*	Calcium/calmodulin-dependent serine protein kinase (CASK) interacting protein 1	2	4.5
3	*CCR5*	Chemokine (C-C motif) receptor 5	5	2.2
X	*CD40LG*	CD40 ligand	6	2.9
1	*CD55*	CD55	11	4.0
7	*CFTR*	Cystic fibrosis transmembrane conductance regulator	3	64.6
6	*CGA*	Glycoprotein hormones α polypeptide	4	2.4
11	*CRACR2B*	Calcium release activated channel regulator 2B	4	2.7
16	*CREBBP*	cAMP response element-binding protein (CREB) binding protein	60	2.7
1	*DARC*	Duffy blood group	1	6.8
6	*DAXX*	Death-domain associated protein	2	4.7
8	*DEPDC6*	DEP domain containing MTOR-interacting protein	112	1.6
14	*DIO2*	Deiodinase iodothyronine type II	11	1.8

14	*DIO3*	Deiodinase iodothyronine type III	0	NA
15	*DYX1C1*	Dyslexia susceptibility 1 candidate 1	38	2.5
4	*EIF4E*	Eukaryotic translation initiation factor 4E	33	1.7
8	*EIF4EBP1*	Eukaryotic translation initiation factor 4E binding protein 1	9	3.9
3	*EPHA3*	Ephrin receptor A3	4	93.7
19	*EPOR*	Erythropoietin receptor	3	3.8
6	*ESR1*	Estrogen receptor α	164	2.5
14	*ESR2*	Estrogen receptor β	51	2.3
20	*FKBP1A*	Tacrolimus binding protein 1A	20	1.5
14	*FOXA1*	Forkhead box A1	3	3.4
13	*FOXO1*	Forkhead box O1	44	2.6
6	*FOXO3*	Forkhead box O3	68	1.9
X	*FOXO4*	Forkhead box O4	6	2.1
19	*FUT2*	Fucosyltransferase 2	6	2.5
X	*G6PD*	Glucose-6-phosphate dehydrogenase	2	8.1
17	*GH1*	Growth hormone 1	1	6.6
5	*GHR*	Growth hormone receptor	100	3.0
20	*GHRH*	Growth hormone releasing hormone	6	1.8
7	*GHRHR*	Growth hormone releasing hormone receptor	14	1.5
11	*HBB*	Haemoglobin β	0	NA
5	*HK3*	Hexokinase 3	13	1.4
11	*HRAS*	Harvey rat sarcoma viral oncogene homolog	5	1.7
12	*IFNg*	Interferon γ	6	1.7
6	*IFNGR1*	Interferon γ receptor 1	7	3.8
21	*IFNGR2*	Interferon γ receptor 2	19	2.1
12	*IGF1*	Insulin-like growth factor 1	32	2.8
15	*IGF1R*	Insulin-like growth factor 1 receptor	166	1.9
7	*IGFBP3*	Insulin-like growth factor binding protein 3	6	2.3
2	*IL1A*	Interleukin 1α	8	2.1
2	*IL1B*	Interleukin 1β	6	2.0
2	*IL1F10*	Interleukin 1 family member 10	10	1.3
2	*IL1RN*	Interleukin 1 receptor antagonist	13	1.6
4	*IL2*	Interleukin 2	3	3.3
X	*IL2RG*	Interleukin 2 receptor γ	2	4.6
5	*IL4*	Interleukin 4	8	1.7

16	*IL4R*	Interleukin 4 receptor	27	2.1
5	*IL5*	Interleukin 5	6	1.2
7	*IL6*	Interleukin 6	2	4.9
8	*IL7*	Interleukin 7	29	2.7
5	*IL7R*	Interleukin 7 receptor	15	1.7
1	*IL10*	Interleukin 10	13	1.8
3	*IL12A*	Interleukin 12A	7	1.7
19	*IL12RB1*	Interleukin 12 receptor β1	14	2.3
5	*IL13*	Interleukin 13	0	NA
6	*IL17A*	Interleukin 17A	6	1.5
2	*IL18RAP*	Interleukin 18 receptor accessory protein	23	1.7
14	*IL25*	Interleukin 25	1	3.6
2	*IL36RN*	Interleukin 36 receptor antagonist	3	3.7
2	*IL37*	Interleukin 37	6	1.8
11	*INS-IGF2*	INS-IGF2 read-through gene	21	1.8
19	*INSR*	Insulin receptor	117	1.6
X	*IRAK1*	Interleukin-1 receptor-associated kinase 1	0	NA
12	*IRAK4*	Interleukin-1 receptor-associated kinase 4	16	2.2
2	*IRS1*	Insulin receptor substrate 1	35	2.1
13	*IRS2*	Insulin receptor substrate 2	23	1.6
9	*JAK2*	Janus kinase 2	53	2.8
2	*KDM3A*	Lysine (K)-specific demethylase 3A	33	1.7
19	*KIR3DL1*	Killer cell immunoglobulin-like receptor, three domains, long cytoplasmic tail 1	3	6.4
19	*KIR3DL3*	Killer cell immunoglobulin-like receptor, three domains, long cytoplasmic tail 3	5	3.4
12	*KMT2D*	Lysine (K)-specific methyltransferase 2D	14	2.4
4	*LEF1*	Lymphoid enhancer-binding factor 1	74	1.7
1	*LEPR*	Leptin receptor	101	2.2
19	*LILRA3*	Leukocyte immunoglobulin-like receptor subfamily A member 3	5	1.9
6	*LIN28B*	Lin-28 homolog B	37	3.5
9	*MAPKAP1*	Mitogen-activated protein kinase associated protein 1	108	2.5
10	*MBL2*	Mannose-binding lectin (protein C) 2	6	1.9
2	*MCM6*	Minichromosome maintenance complex component 6	3	12.3

20	*MCM8*	Minichromosome maintenance 8 homologous recombination repair factor	23	2.2
1	*MTOR*	Mechanistic target of rapamycin	81	2.0
3	*MYD88*	Myeloid differentiation primary response 88	5	1.9
11	*MYO7A*	Myosin VIIA	1	87.0
8	*NCOA2*	Nuclear receptor coactivator 2	123	2.4
14	*NFKBIA*	Nuclear factor of κ light polypeptide gene enhancer in B-cells inhibitor α	7	1.2
8	*NKX3-1*	Homeobox Nkx-3.1	3	3.1
1	*NR0B2*	Nuclear receptor subfamily 0 group B member 2	2	3.8
19	*NR1H2*	Nuclear receptor subfamily 1 group H member 2	6	1.9
11	*NR1H3*	Nuclear receptor subfamily 1 group H member 3	8	3.1
12	*PARPBP*	Poly (ADP-ribose) polymerase 1 binding protein	2	38.7
2	*PDK1*	Pyruvate dehydrogenase kinase isozyme 1	5	13.9
7	*PDK4*	Pyruvate dehydrogenase kinase isozyme 4	15	1.2
16	*PDPK1*	3-phosphoinositide dependent protein kinase 1	11	6.4
19	*PGPEP1*	Pyroglutamyl-peptidase I	6	4.9
3	*PIK3CB*	Phosphatidylinositol-4,5-bisphosphate 3-kinase catalytic subunit β	43	2.5
5	*PIK3R1*	Phosphoinositide-3-kinase regulatory subunit 1	40	2.0
12	*PMCH*	Pro-melanin-concentrating hormone	3	2.1
5	*PPARGC1B*	Peroxisome proliferator-activated receptor γ coactivator 1β	73	1.7
22	*PRR5*	Proline rich 5	44	1.7
10	*PTEN*	Phosphatase and tensin homolog	17	6.5
20	*PTPN1*	Protein tyrosine phosphatase non-receptor type 1	34	2.3
3	*PTX3*	Pentraxin 3	6	4.2
17	*RARA*	Retinoic acid receptor α	10	5.3
22	*RBFOX2*	RNA binding protein fox-1 homolog 2	92	3.1

5	*RICTOR*	RPTOR independent companion of MTOR complex 2	52	2.7
8	*RIPK2*	Receptor-interacting serine-threonine kinase 2	20	1.9
14	*RNASE3*	Ribonuclease A family 3	1	5.9
17	*RPS6KB1*	Ribosomal protein S6 kinase polypeptide 1	21	3.0
17	*RPTOR*	Regulatory associated protein of MTOR complex 1	235	1.8
18	*SLC14A1*	Urea transporter member 1 (Kidd blood group)	24	1.4
4	*SPP1*	Secreted phosphoprotein 1	2	3.9
3	*SST*	Somatostatin	3	2.2
22	*SSTR3*	Somatostatin receptor 3	6	1.9
17	*STAT5A*	Signal transducer and activator of transcription 5A	12	2.4
17	*STAT5B*	Signal transducer and activator of transcription 5B	31	2.7
19	*SUV420H2*	Suppressor of variegation 4-20 homolog 2	10	1.3
6	*SYCP2L*	Synaptonemal complex protein 2-like	66	1.4
19	*TCF3*	Transcription factor 3	18	2.4
19	*TGFB1*	Transforming growth factor β1	11	2.6
17	*THRA*	Thyroid hormone receptor α	16	2.3
1	*THRAP3*	Thyroid hormone receptor associated protein 3	26	3.3
3	*THRB*	Thyroid hormone receptor β	179	2.1
19	*TICAM1*	Toll-like receptor adaptor molecule 1	10	2.1
5	*TICAM2*	Toll-like receptor adaptor molecule 2	34	1.5
11	*TIRAP*	Toll-interleukin 1 receptor domain containing adaptor protein	8	2.1
4	*TLR1*	Toll-like receptor 1	4	3.4
4	*TLR2*	Toll-like receptor 2	19	2.0
4	*TLR3*	Toll-like receptor 3	17	1.2
9	*TLR4*	Toll-like receptor 4	33	0.9
1	TLR5	Toll-like receptor 5	20	1.9
4	*TLR6*	Toll-like receptor 6	4	1.9
X	*TLR7*	Toll-like receptor 7	11	2.6
X	*TLR8*	Toll-like receptor 8	9	2.4
3	*TLR9*	Toll-like receptor 9	2	5.0

4	*TLR10*	Toll-like receptor 10	11	1.4
6	*TNF*	Tumour necrosis factor	1	7.8
19	*TOMM40*	Translocase of outer mitochondrial membrane 40 homolog	11	1.6
9	*TSC1*	Tuberous sclerosis 1	22	2.6
16	*TSC2*	Tuberous sclerosis 2	18	2.5
14	*TSHR*	Thyroid stimulating hormone receptor	104	1.9
5	*UIMC1*	Ubiquitin interaction motif containing 1	51	2.1
22	*USP41*	Ubiquitin specific peptidase 41	1	4.0

Candidate genes included in the analyses are given with the number of SNPs associated to these genes included in the analyses. SNPs included as selectively neutral controls are not included in this table. An impression of the density of SNPs per gene is given by the average number of 1000 base pairs (kb) between the SNPs included in the analyses. The sizes of the gene regions were derived from Genome Reference Consortium Human Build 37 patch release 1 (GRCh37.p1).

Supplementary Table 2. SNPs with the lowest *p* values for the association with survival between newborns and old individuals aged 60 years or over

Chromosome	Gene symbol	SNP	MAF	OR	*p* value
14	*AKT1*	rs61758466	0.14	0.73	2.88×10^{-4}
13	*IRS2*	rs2026816	0.32	0.81	4.55×10^{-4}
21	*CLDN14**	rs2835370	0.49	1.21	5.07×10^{-4}
15	*IGF1R*	rs2684796	0.41	0.83	7.17×10^{-4}
12	*PARPBP*	rs17032311	0.01	2.46	9.41×10^{-4}
3	*THRB*	rs75500315	0.02	2.08	1.18×10^{-3}
7	*IL6*	rs2069842	0.11	0.75	1.41×10^{-3}
6	*SYCP2L*	rs9368446	0.03	1.62	1.44×10^{-3}
19	*TCF3*	rs2353219	0.20	0.79	1.54×10^{-3}
16	*CLEC3A**	rs430046	0.27	0.82	1.78×10^{-3}

The analysis was adjusted for sex. MAF: minor allele frequency. OR: odds ratio. * These SNPs were included as selectively neutral controls. The level of significance is 1.23×10^{-5}.

Supplementary Table 3. SNPs with the lowest p values for the association with survival between newborns and middle-aged adults of fertile ages from 20 through 44 years

Chromosome	Gene symbol	SNP	MAF	OR	*p* value
6	*SYCP2L*	rs75788404	0.09	0.70	4.88×10^{-4}
6	*ESR1*	rs1293936	0.46	1.22	6.10×10^{-4}
13	*IRS2*	rs2026816	0.32	0.81	7.67×10^{-4}
7	*IL6*	rs2069842	0.11	0.74	9.15×10^{-4}
9	*JAK2*	rs60763646	0.10	0.74	1.27×10^{-3}
8	*RIPK2*	rs2158131	0.41	1.21	1.36×10^{-3}
9	*JAK2*	rs4372063	0.03	1.68	1.68×10^{-3}
6	*SYCP2L*	rs57579421	0.08	0.73	1.86×10^{-3}
8	*RIPK2*	rs16900627	0.42	1.20	2.05×10^{-3}
8	*RIPK2*	rs73291472	0.04	0.63	2.16×10^{-3}

The analysis was adjusted for sex. MAF: minor allele frequency. OR: odds ratio. The level of significance is 1.23×10^{-5}.

Supplementary Table 4. SNPs with the lowest *p* values for the association with survival between middle-aged adults of fertile ages from 20 through 44 years and old individuals aged 60 years or over

Chromosome	Gene symbol	SNP	MAF	OR	*p* value
9	*TLR4*	rs10818073	0.03	1.90	4.80×10^{-4}
5	*UIMC1*	rs75988289	0.03	1.70	8.90×10^{-4}
13	*ARHGEF7*	rs74691269	0.10	1.38	9.50×10^{-4}
8	*NCOA2*	rs6983366	0.02	0.52	1.71×10^{-3}
3	*THRB*	rs6785472	0.16	1.30	1.78×10^{-3}
13	*ARHGEF7*	rs79847747	0.09	1.36	2.09×10^{-3}
8	*NCOA2*	rs57293541	0.06	0.68	2.29×10^{-3}
3	*THRB*	rs6550862	0.15	1.28	2.49×10^{-3}
5	*GHR*	rs73085419	0.11	1.31	2.79×10^{-3}
1	*CD55*	rs75882193	0.02	1.92	2.85×10^{-3}

The analysis was adjusted for sex. MAF: minor allele frequency. OR: odds ratio. The level of significance is 1.23×10^{-5}.

Supplementary Table 5. SNPs with the lowest *p* values for the association with observed fertility in middle-aged women of fertile ages from 20 through 44 years

Chromosome	Gene symbol	SNP	MAF	OR	*p* value
19	*TGFB1*	rs4803455	0.38	0.78	5.23×10^{-4}
3	*THRB*	rs1505301	0.44	0.79	9.19×10^{-4}
17	*INPP5K**	rs1879488	0.23	1.27	1.68×10^{-3}
22	*RBFOX2*	rs916333	0.12	1.32	2.73×10^{-3}
8	*NCOA2*	rs16936902	0.12	1.32	3.25×10^{-3}
22	*PRR5*	rs2349642	0.40	1.22	3.47×10^{-3}
22	*PRR5*	rs2097535	0.40	1.22	3.81×10^{-3}
17	*STAT5B*	rs9897531	0.08	1.39	4.07×10^{-3}
2	*PABPCP2*	rs10496971	0.07	1.38	4.64×10^{-3}
1	*ARID1A*	rs4589135	0.42	1.22	5.05×10^{-3}

* This SNP was included as a selectively neutral control. MAF: minor allele frequency. OR: odds ratio. The level of significance is 1.61×10^{-5}.

Supplementary Table 6. SNPs with the lowest *p* values for the association with reported fertility in postmenopausal women aged 45 years and older

Chromosome	Gene symbol	SNP	MAF	OR	*p* value
2	*IL1RN*	rs4251987	0.02	4.95	3.50×10^{-4}
15	*DYX1C1*	rs79593205	0.04	2.77	9.97×10^{-4}
20	*PTPN1*	rs77833095	0.05	0.41	1.04×10^{-3}
13	*IRS2*	rs74886884	0.01	7.17	1.41×10^{-3}
6	*FOXO3*	rs75621079	0.10	1.90	1.42×10^{-3}
17	*STAT5B*	rs56938023	0.25	0.64	2.25×10^{-3}
6	*ESR1*	rs17082104	0.13	1.73	2.68×10^{-3}
15	*IGF1R*	rs75090574	0.04	2.61	2.71×10^{-3}
19	*APOE*	rs1081101	0.06	0.46	3.11×10^{-3}
3	*THRB*	rs62255856	0.15	1.63	3.45×10^{-3}

MAF: minor allele frequency. OR: odds ratio. The level of significance is 1.23×10^{-5}.

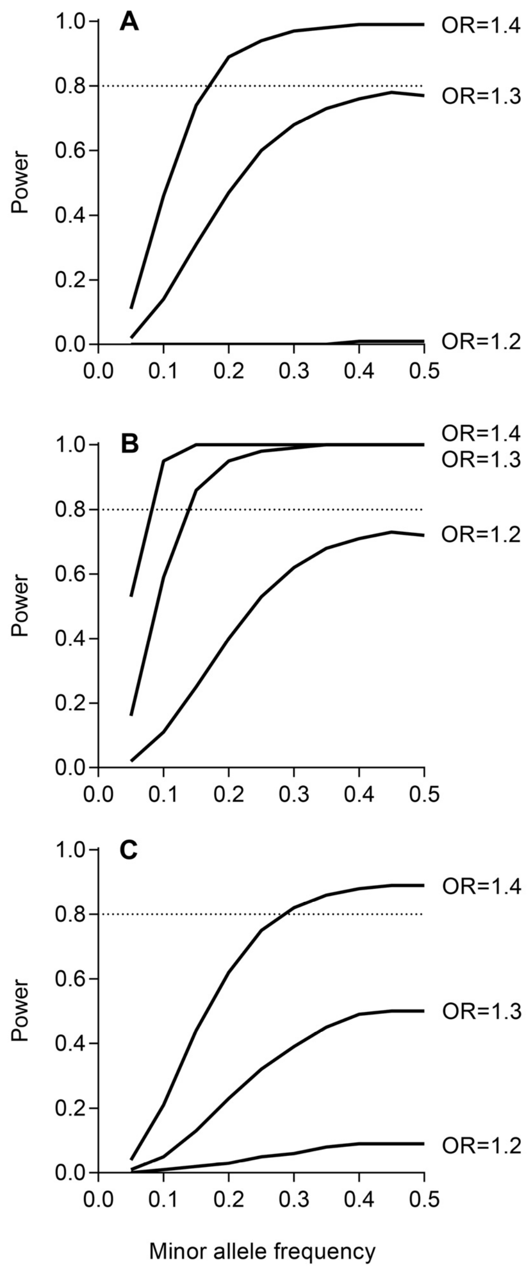

Supplementary Figure 1. A post hoc calculation of this study's power, depending on the strength of the SNP's effect and the minor allele frequency. The effect is expressed as an odds ratio (OR) determined using an additive logistic regression model assessing the associations of SNPs with survival between newborns and old individuals aged 60 and over (**A**), between newborns and middle-age adults of fertile ages from 20 through 44 years (**B**), and between middle-aged adults of fertile ages from 20 through 44 years and old individuals aged 60 years or over (**C**).
